# [^18^F]FDG PET/CT–Avid Discordant Volume as a Biomarker in Patients with Gastroenteropancreatic Neuroendocrine Neoplasms: A Multicenter Study

**DOI:** 10.2967/jnumed.123.266346

**Published:** 2024-02

**Authors:** David L. Chan, Aimee R. Hayes, Ioannis Karfis, Alice Conner, Magdalena Mileva, Elizabeth Bernard, Geoffrey Schembri, Shaunak Navalkissoor, Gopinath Gnanasegaran, Nick Pavlakis, Clémentine Marin, Bruno Vanderlinden, Patrick Flamen, Paul Roach, Martyn E. Caplin, Christos Toumpanakis, Dale L. Bailey

**Affiliations:** 1 Faculty of Medicine and Health, University of Sydney, Sydney, New South Wales, Australia;; 2 Medical Oncology, ENETS Centre of Excellence, Royal North Shore Hospital, Sydney, New South Wales, Australia;; 3 Neuroendocrine Tumour Unit, ENETS Centre of Excellence, Royal Free Hospital, London, United Kingdom;; 4 Nuclear Medicine Department, Institut Jules Bordet, ENETS Centre of Excellence, Hôpital Universitaire de Bruxelles, Université Libre de Bruxelles, Brussels, Belgium;; 5 Nuclear Medicine, ENETS Centre of Excellence, Royal North Shore Hospital, Sydney, New South Wales, Australia;; 6 Nuclear Medicine, ENETS Centre of Excellence, Royal Free Hospital, London, United Kingdom; and; 7 Medical Physics Department, Institut Jules Bordet, ENETS Centre of Excellence, Hôpital Universitaire de Bruxelles, Université Libre de Bruxelles, Brussels, Belgium

**Keywords:** prognostic biomarker, discordance, [^18^F]FDG, dual PET, tumor volume

## Abstract

[^18^F]FDG PET/CT and [^68^Ga]Ga-DOTATATE PET/CT are both used to predict tumor biology in neuroendocrine neoplasms. Although the presence of discordant ([^18^F]FDG-avid/non–[^68^Ga]Ga-DOTATATE–avid) disease predicts poor prognosis, the significance of the volume of such discordant disease remains undetermined. The aim of this study is to investigate discordant tumor volume as a potential biomarker in patients with advanced gastroenteropancreatic neuroendocrine neoplasms (GEPNENs). **Methods:** A multicenter retrospective study in patients with advanced GEPNENs and paired [^18^F]FDG and [^68^Ga]Ga-DOTATATE PET/CT no more than 85 d apart was conducted. Patients with discordant disease were identified by the NETPET score, and discordant lesions were contoured with a flat [^18^F]FDG SUV cutoff of 4. The primary variable of interest was the total discordant volume (TDV), which was the sum of the volumes of discordant lesions. Patients were dichotomized into high- and low-TDV cohorts by the median value. The primary endpoint was overall survival. **Results:** In total, 44 patients were included (50% men; median age, 60 y), with primary cancers in the pancreas (45%), small bowel (23%), colon (20%), and other (12%). Of the patients, 5% had grade 1 disease, 48% had grade 2 disease, and 48% had grade 3 disease (24% well differentiated, 67% poorly differentiated, 10% unknown within the grade 3 cohort). The overall median survival was 14.1 mo. Overall survival was longer in the low-TDV cohort than in the high-TDV cohort (median volume, 43.7 cm^3^; survival time, 23.8 mo vs. 9.4 mo; hazard ratio, 0.466 [95% CI, 0.229–0.948]; *P* = 0.0221). Patients with no more than 2 discordant intrahepatic lesions survived longer than those with 2 or more lesions (31.8 mo vs. 10.2 mo, respectively; hazard ratio, 0.389 [95% CI, 0.194–0.779]; *P* = 0.0049). **Conclusion:** TDV is a potential prognostic biomarker in GEPNENs and should be investigated in future neuroendocrine neoplasm trials.

## Visual Abstract

**Figure:**
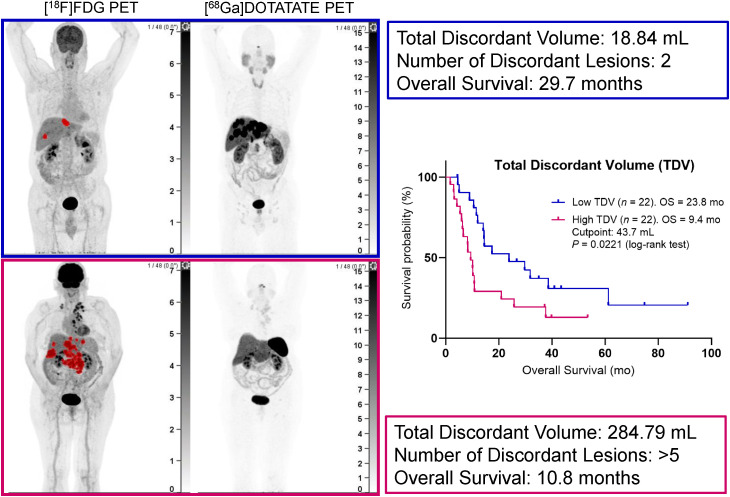

Neuroendocrine neoplasms (NENs) are a heterogeneous group of tumors that may produce vasoactive hormones. Although NENs are currently uncommon, recent data show that the incidence and prevalence of NENs are significantly increasing (1). Because of the insidious and nebulous symptoms of NENs, many patients present with disease at an advanced stage (2) and are treated with systemic therapy without curative intent. Prognostication in these patients is of the utmost importance as it helps patients make difficult life decisions while assisting physicians in the selection of the optimal systemic therapy. Patients with low-grade disease tend to have poor response rates to chemotherapy (3) and are instead preferentially treated with somatostatin analogs, targeted therapies, and peptide receptor radionuclide therapy. Conversely, patients with high-grade disease exhibit poor response rates to the aforementioned treatments and are generally treated with chemotherapy (4).

Diagnosing a patient with low- or high-grade disease requires histologic analysis of biopsied or resected tissue. However, tissue is often taken from a single lesion, in a disease in which patients commonly have multiple tumor sites (5) that are biologically heterogeneous. The use of repeat tissue sampling is limited by the risk of complications, and the lack of tissue from a fine-needle biopsy limits accurate grade determination (6). This potentially results in grading misclassification, resulting in suboptimal therapy selection. Finally, the histologic grade alone cannot explain the observed clinical heterogeneity in patients with NENs. Therefore, there is a pressing need for repeatable and ideally noninvasive biomarkers to accurately stratify NEN tumor behavior in vivo.

PET/CT has entered the mainstream clinical practice in NEN imaging. Particularly, the combined interpretation of [^68^Ga]Ga-DOTATATE PET and [^18^F]FDG PET (dual PET) is emerging as a highly informative clinical tool in patients with metastatic gastroenteropancreatic NENs (GEPNENs) (7). [^68^Ga]Ga-DOTATATE PET utilizes somatostatin receptors (particularly subtype 2) as targets, which are overexpressed in well-differentiated and typically low-grade NENs. Accordingly, high [^68^Ga]Ga-DOTATATE avidity is associated with lower-grade, well-differentiated histology and better prognosis (8). [^18^F]FDG PET avidity reflects glycolysis and hence areas of increased metabolic activity. This avidity is associated with higher-grade, poorly differentiated histology and poorer prognosis (9). We previously developed a 5-grade scoring system (known colloquially as the NETPET score) (10) to succinctly capture the complexity of dual PET findings to guide prognostication. We demonstrated that patients with [^68^Ga]Ga-DOTATATE–avid disease only (without any [^18^F]FDG avidity) have the best prognosis, that those with concordant disease ([^18^F]FDG avidity corresponding with [^68^Ga]Ga-DOTATATE avidity) have intermediate prognosis, and that those with any degree of discordant disease have the worst prognosis (11). This work suggests that discordant disease may be considered a poor prognostic marker. Although research investigating [^68^Ga]Ga-DOTATATE PET or [^18^F]FDG PET as a prognostic biomarker is promising, most studies (including those cited above) have largely concentrated on point measures or SUVs (SUV_max _and SUV_peak_), without incorporating tumor volume as a separate prognostic biomarker.

Several studies have established the prognostic value of PET-avid tumor volume on [^18^F]FDG PET and [^68^Ga]Ga-DOTATATE PET separately (12–14). These studies showed that increased tumor volume on either scan is associated with an increasingly worse prognosis, but these findings have not yet been applied to dual PET imaging.

Given the lack of data to date integrating the concepts of discordant disease and tumor volume, we sought to investigate the discordant tumor volume as a potential new imaging-based biomarker. We conducted a retrospective analysis to investigate the prognostic impact of this discordant tumor volume in patients with advanced NENs.

## MATERIALS AND METHODS

### Patient Cohort

Consecutive patients with a histologically confirmed GEPNEN and dual PET/CT scans no more than 85 d apart were identified on retrospective review across 3 institutions, as previously published (10*,*^15^*,*^16^). Of these patients, only those with a NETPET score of P4b/P5 (indicating the presence of discordant disease) were selected for analysis in this study. A follow-up period of at least 30 d was necessary for inclusion in this study.

This study was approved by the institutional review boards of the Northern Sydney Local Health District Human Research Ethics Committee (2019/ETH14060), the Royal Free Hospital NHS Foundation Trust Quality Governance and Clinical Audit Committee, and the Ethics Committee of Jules Bordet Institute (CE2531). Each committee waived the need for informed consent.

### PET-Based Analysis

Imaging data were acquired as reported previously (11) on comparable current-generation PET/CT scanners at each center with time-of-flight capabilities as whole-body scans (vertex of skull to mid thigh). Physicians had full access to software tools and clinical history at the time of the scan, reflecting the normal clinical practice. Dual PET scans were anatomically coregistered and displayed on a dedicated nuclear medicine–reporting workstation in transverse, coronal, and sagittal planes with rotating maximum-intensity projection cine images. All software tools were available for use, including window adjustment, color tables, and distance-defining calipers. Positivity on each scan was defined as an uptake visually greater than background uptake for the specific tissue surrounding the region of interest. Experienced nuclear medicine physicians reviewed each pair of PET scans qualitatively to derive the NETPET score (10).

**FIGURE 1.**
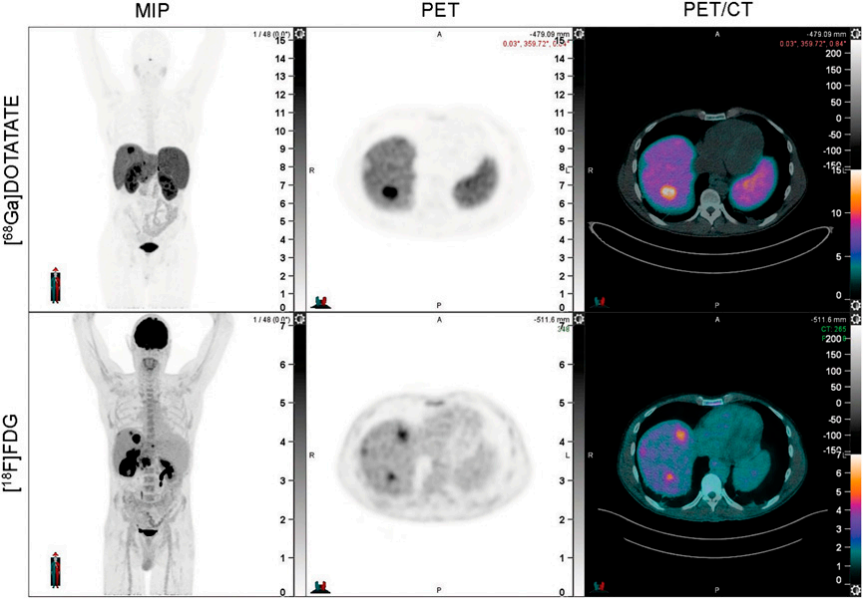
Example of discordant lesion, which is [^18^F]FDG PET–positive but [^68^Ga]Ga-DOTATATE PET–negative. From left to right, panels reflect maximum-intensity projection (MIP) image, PET, and PET/CT fusion.

Discordant lesions showing [^18^F]FDG avidity but no [^68^Ga]Ga-DOTATATE avidity were identified by experienced nuclear medicine physicians on simultaneous review of both scans. The images were manually contoured on the [^18^F]FDG PET using a flat threshold SUV of 4 (MIM software, version 7.2.7; MIM Software Inc.). An example of a discordant lesion can be seen in Figure 1. Imaging variables of interest included SUV_max_, SUV_mean_, metabolic tumor volume, total lesion glycolysis, and the number and location of discordant lesions.

### Clinicopathologic Variables

Clinical data were collected at each site by a retrospective chart review. Age at diagnosis, sex, primary site of GEPNEN, histologic grade (by World Health Organization 2017–2019 criteria) (6), the presence or absence of extrahepatic disease, and clinical outcomes were recorded.

The primary endpoint of this study was overall survival. Overall survival was calculated from the latter of the dual PET scans to the date of death, with data censored at the last known follow-up.

### Statistical Analysis

The total discordant volume (TDV) of all lesions, the discordant metastatic lesion volume, and the discordant primary volume (in patients with tumors > 0 cm^3^) were dichotomized by the median value of each variable. Similarly, the [^18^F]FDG SUV_mean_ and SUV_max_ across all discordant lesions in each patient were calculated, and patients were dichotomized by their respective median values. Patients were also dichotomized by the presence of no more than 2 or more than 2 total discordant lesions, and no more than 2 or more than 2 discordant intrahepatic lesions as exploratory analyses (with 2 as an arbitrarily chosen cutoff). The overall survival of the resulting 2 groups from each dichotomization was compared using the log-rank test, with an α of 0.05 applied to the primary outcome measurement of TDV and Bonferroni adjustment used for all secondary outcomes.

Cohorts formed by histologic grade and by differentiation status (in patients with grade 3 disease only) were assessed using the log-rank test in groups with *n* greater than 5. This resulted in the exclusion of grade 1 patients from this analysis. The TDVs of grade 2 and grade 3 patients were additionally compared using an unpaired *t* test, with TDV as a continuous variable. Multivariate analysis was not performed because of the relatively small number of patients in this cohort.

## RESULTS

In total, 44 patients with discordant disease were identified from the report by Chan et al. (NETPET P4b/P5) (11) across 3 participating centers in Sydney, London, and Brussels. The median age at diagnosis was 60 y, and 50% of the patients were men (*n* = 22/44). Scans were performed a maximum of 85 d apart, with a median scan interval of 8 d and an interquartile range of 1.75–22.5 d. No patients had progressive disease between the 2 scans. The median mitotic count was 3.5 per 2 mm^2^, and the median Ki-67 index was 15%. The most common primary sites were the pancreas (45%, *n* = 20/44), small bowel (23%, *n* = 10/44), and colon (20%, *n* = 9/44). Using the World Health Organization 2019 criteria, we determined that 5% of patients were grade 1 (*n* = 2/44), 48% were grade 2 (*n* = 21/44), and 48% were grade 3 (*n* = 21/44). Within the grade 3 cohort, 24% were well differentiated (*n* = 5/21), 67% were poorly differentiated (*n* = 14/21), and 10% had unknown differentiation (*n* = 2/21). The median follow-up period of surviving patients was 40 mo. The median overall survival of the cohort was 14.1 mo. Summarized cohort demographic data are available in Table 1.

**TABLE 1.** Cohort Summary Characteristics

Characteristic	Patients (*n* = 44)
Sex	
Male	22 (50%)
Female	22 (50%)
Primary site of tumor	
Pancreas	20 (45%)
Midgut	10 (23%)
Colorectal	9 (20%)
Other	3 (12%)
Histologic grade	
Grade 1	2 (5%)
Grade 2	21 (48%)
Grade 3 (differentiation)	21 (48%; 24% well differentiated, 67% poorly differentiated, 10% unknown)
Extrahepatic disease	
Yes	34 (77%)
No	10 (23%)
Functional disease	
Yes	9 (20%)
No	26 (59%)
Unknown	9 (20%)

Mean age at diagnosis was 60 y (range, 24–82 y).

Regarding the distribution of discordant lesions, 15 patients (34%) had discordant disease confined to the liver, 9 patients (20%) had discordant disease in extrahepatic sites only, and 20 patients (45%) had discordant disease both inside and outside the liver. The median discordant tumor volume used for dichotomization was 43.7 cm^3^. The median TDVs of the low-TDV group and high-TDV group were 6.75 and 137.22 cm^3^, respectively. There was no statistically significant difference in post-PET therapy choices between the 2 groups.

**FIGURE 2.**
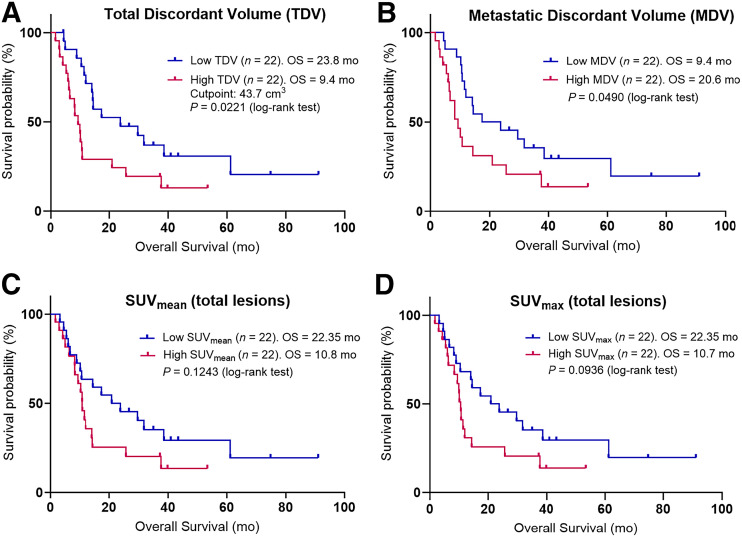
Kaplan–Meier curves for overall survival (OS) are shown for each variable. Patients were dichotomized into 2 groups based on median value of TDV (A) (43.7 cm^3^), metastatic discordant volume (B) (30.2 cm^3^), SUV_mean_ of all discordant lesions (C) (dichotomization SUV, 6), and SUV_max_ of all discordant lesions (D) (dichotomization SUV, 12.6).

Overall survival was longer in the cohort with low TDV than in the cohort with high TDV, with median survival of 23.8 mo versus 9.4 mo, respectively (cohort median volume, 43.7 cm^3^; hazard ratio, 0.466 [95% CI, 0.229–0.948]; *P* = 0.0221; Fig. 2A).

**FIGURE 3.**
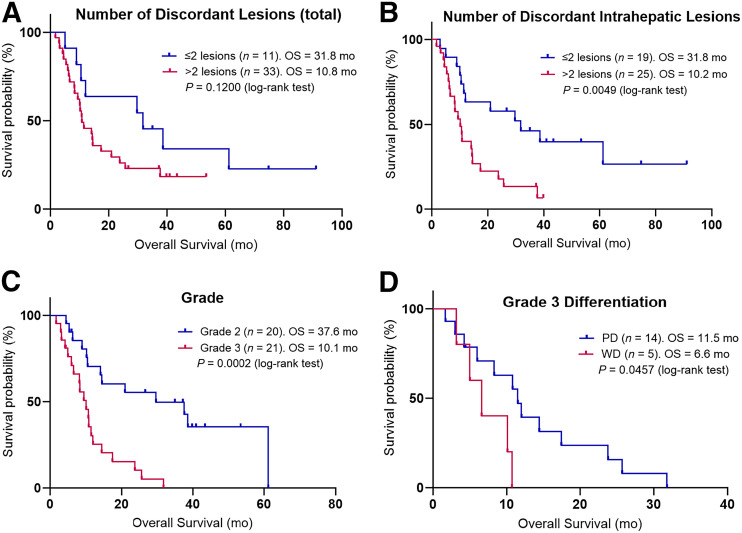
Kaplan–Meier curves for overall survival (OS) are shown for each variable. Patients were dichotomized by number of discordant lesions (A) (≤2 or >2), number of discordant intrahepatic lesions (B) (≤2 or >2), tumor grade (C) (grade 1 patients were excluded as *n* < 5), and differentiation of grade 3 patients (D) (well differentiated [WD] and poorly differentiated [PD]).

**FIGURE 4.**
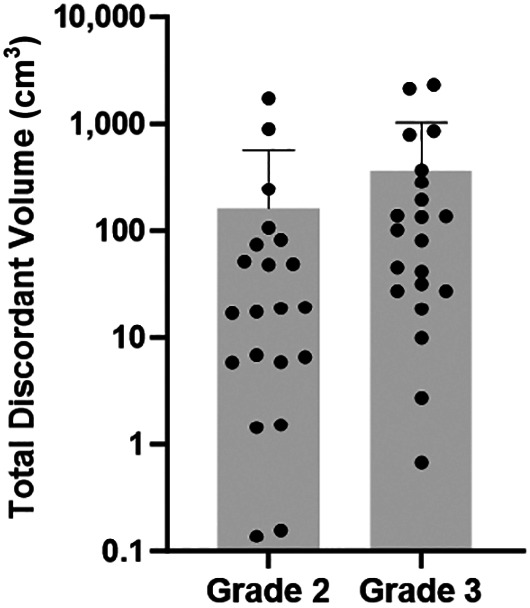
TDV by histologic grade. Groups (*n* = 21 each) were not significantly different (grade 2 mean, 166.4 cm^3^; grade 3 mean, 367.3 cm^3^; *P* = 0.2279, unpaired *t* test). Patients with grade 1 disease were excluded from analysis because of small sample (*n* < 5). Gray bars represent mean value, and error bars represent SD.

Using a Bonferroni adjustment, the adjusted α-value for secondary outcomes had a *P* value of 0.006. There was no significant difference in overall survival when using the Bonferroni adjustment and dichotomizing by metastatic volume (*P* = 0.049; Fig. 2B) or primary volume (*P* = 0.1724). The SUV_mean_ and SUV_max_ across all lesions were not significantly associated with overall survival on a patient-level basis (*P* = 0.1243 and 0.0936, respectively; Figs. 2C and 2D). The total number of discordant lesions of no more than 2 or more than 2 was not significantly associated with survival (*P* = 0.1200; Fig. 3A); however, patients with a maximum of 2 discordant intrahepatic lesions had significantly longer survival than those with more than 2 such lesions (31.8 mo vs. 10.2 mo; hazard ratio, 0.389 [95% CI, 0.194–0.779]; *P* = 0.0049; Fig. 3B). There was a significant difference between the survival of grade 2 and the survival of grade 3 patients (*P* = 0.0002; Fig. 3C) but not between those with poorly differentiated and well-differentiated grade 3 histology (*P* = 0.0457). Given the focus on [^18^F]FDG PET over [^68^Ga]Ga-DOTATATE PET in grade 3 poorly differentiated neuroendocrine carcinoma, we conducted a sensitivity analysis restricting the cohort of patients to those with well-differentiated disease alone (i.e., grade 1, grade 2, and grade 3 well-differentiated NENs) and redichotomizing the median TDV of this new cohort (19.1 cm^3^). There was no significant difference in the overall survival of low- versus high-TDV patients in this subgroup (38.6 and 10.7 mo, respectively; *P* = 0.1373), likely due to the lower number of patients. Detailed statistical results for the overall survival of patients with these variables are presented in Table 2. There was no significant correlation between the TDV of grade 2 and grade 3 patients (*P* = 0.2279; Fig. 4).

**TABLE 2.** Statistical Results for Each Variable Using Log-Rank Test

Variable	Value used to dichotomize cohort	Median overall survival (mo) of group below/above dichotomization	Hazard ratio*	*P*
TDV	43.7 cm^3^	23.8/9.4	0.466 (0.229–0.948)	0.0221
Metastatic discordant volume	30.2 cm^3^	20.6/9.4	0.516 (0.253–1.053)	0.0490
Primary discordant volume	12.1 cm^3^	11.5/10.7	1.272 (0.380–4.261)	0.1724
SUV_mean_	6	22.35/10.8	0.596 (0.296–1.200)	0.1243
SUV_max_	12.6	22.35/10.7	0.569 (0.282–1.151)	0.0930
Discordant lesions	≤2 lesions, >2 lesions	31.8/10.8	0.550 (0.271–1.116)	0.1200
Intrahepatic lesions	≤2 lesions, >2 lesions	31.8/10.2	0.389 (0.194–0.779)	0.0049
Grade	Grade 2, grade 3	29.7/10.1	0.303 (0.146–0.631)	0.0002
Grade 3 differentiation	Well differentiated, poorly differentiated	6.6/11.5	2.568 (0.651–10.130)	0.0457

* Hazard ratio with 95% CI.

## DISCUSSION

**FIGURE 5.**
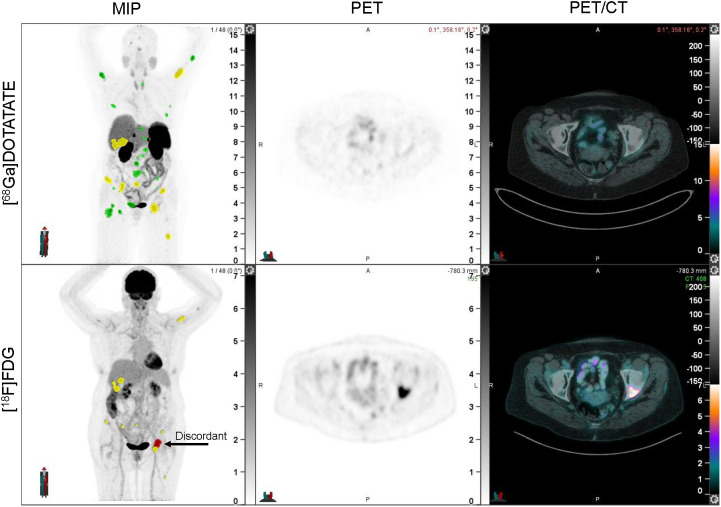
Example patient in low-TDV group with TDV of 9.91 cm^3^. Patient has 31 [^68^Ga]Ga-DOTATATE–positive lesions, 21 of which are avid on [^68^Ga]Ga-DOTATATE only (green) and 10 of which have corresponding [^18^F]FDG avidity (yellow). Of 11 [^18^F]FDG-avid lesions with SUV of at least 4, 1 is discordant ([^68^Ga]Ga-DOTATATE–negative, in red). From left to right, panels reflect the maximum-intensity projection (MIP) image, PET, and PET/CT fusion.

The current study demonstrates for the first time, to our knowledge, that higher volumes of discordant disease on dual PET imaging are a prognostic biomarker in patients with metastatic GEPNENs. Although our group has previously shown that the presence of discordant disease predicts poor prognosis (P5 cohort (11)), the current study further stratifies these patients into cohorts of high- and low-volume discordant disease. Although prior studies have looked at tumor volume on [^68^Ga]Ga-DOTATATE PET and [^18^F]FDG PET in isolation (10*,*^14^), the volumetric implications from overlaying the 2 scans have not previously been considered. For instance, the patient in Figure 5 displays multiple [^68^Ga]Ga-DOTATATE–avid and [^18^F]FDG-avid lesions. However, dual PET analysis highlights that a low volume of this disease is discordant. We note that both flat cutoffs and adaptive cutoffs have been used in prior studies (12–14). We elected to use a flat cutoff for the current study, in keeping with previous work investigating [^18^F]FDG PET (12). We note that dual PET has been used successfully in the prediction of tumor aggressiveness and clinical outcomes in patients with resectable pancreatic NENs (17*,*^18^).

Patients were included in this multicenter study regardless of their age, histologic grade, or GEPNEN primary site, resulting in a representative real-world cohort. Our cohort contained 2 patients with grade 1 histology who also demonstrated discordant disease. This is not entirely unexpected, as a significant minority of patients diagnosed with grade 1 histology (on initial biopsy) exhibit [^18^F]FDG avidity (19). Our findings reinforce the role of dual PET in metastatic GEPNENs, even in a low-grade population. Although analysis of the well-differentiated cohort did not show a significant association between TDV and overall survival, the smaller numbers in this cohort might explain the lack of statistical significance, as we note that the 28-mo numeric difference in overall survival between high- and low-TDV cohorts remains clinically significant and mirrored that of the overall population. The exploratory analysis showing possibly poorer prognosis for patients with a higher number of discordant intrahepatic lesions may reflect the difficulty in giving liver-directed therapy to such patients. Whether this is true may be investigated in future studies. The findings may be because patients with a high number of discordant intrahepatic lesions may not be effectively targeted by liver-directed therapy, meaning that only systemic therapies are available to keep this more biologically aggressive disease under control. However, given the exploratory nature of such analyses, no firm conclusions regarding the use of these metrics in clinical practice can be made at this point.

The findings of this study carry practical implications for the practicing NEN physician. Patients with low-volume discordant disease have a significantly better prognosis than those with high-volume disease but may require a variety of treatment approaches. For example, patients with 1–2 oligodiscordant metastases in the setting of mostly [^68^Ga]Ga-DOTATATE–avid disease may still benefit from peptide receptor radionuclide therapy, but with the addition of a simultaneous anatomically targeted therapy (e.g., stereotactic body radiotherapy) to specifically target low-volume discordant lesions. This is particularly the case with discordant disease limited to the liver, for which liver-directed therapy in addition to simultaneous therapy (perhaps even peptide receptor radionuclide therapy) may deal effectively with non–[^68^Ga]Ga-DOTATATE–avid NEN lesions. The better prognosis of this subgroup justifies the complexity, health resource use, and potential complications of this combination treatment. On the other hand, patients with high-volume discordant disease have a significantly shorter survival (median survival was 9.4 mo in the current cohort), suggesting that more aggressive treatments should be considered. Given the significant amount of non–[^68^Ga]Ga-DOTATATE–avid aggressive disease in this cohort, patients should most likely be treated with chemotherapy and be prioritized for clinical trials because of their anticipated poor outcome.

Our study had the strength of pooling data across multiple centers using similar scanners, paving the way for clinical translation to practice across multiple NEN practices globally. This collaboration allowed a significant number of patients to be identified in an uncommon subset of an uncommon tumor. We prespecified the study methodology to ensure that scan analysis was conducted similarly across geographically disparate areas. We acknowledge the limitations of the current study arising from the retrospective design and the relatively small sample size. As mentioned above, whereas the 44 patients identified in the present study represent a significant cohort in discordant advanced NENs, multivariate analysis is not feasible in a cohort of this size.

**FIGURE 6.**
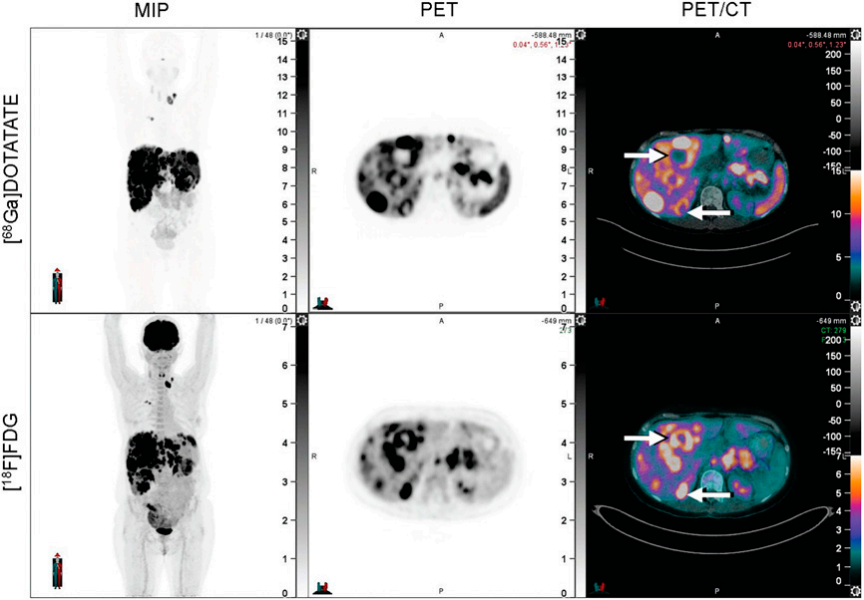
Example of heterogeneous discordance. Arrows highlight areas of [^18^F]FDG discordance in a lesion that otherwise displays concordant [^68^Ga]Ga-DOTATATE and [^18^F]FDG avidity. From left to right, panels reflect the maximum-intensity projection (MIP) image, PET, and PET/CT fusion.

The current study suggests several paths forward for future research. Although the NETPET system (10) assumes lesion-level homogeneous uptake (or lack of uptake), future studies may elucidate the prognostic significance of lesions with heterogeneous uptake within the tumor. Particularly, lesions with heterogeneous discordance (Fig. 6) should be explored to determine whether a small amount of discordance within an otherwise [^68^Ga]Ga-DOTATATE–avid lesion truly represents discordant disease and carries its associated poor prognosis. Additionally, an analysis of the lesions with [^68^Ga]Ga-DOTATATE avidity but not [^18^F]FDG avidity may yield additional information (e.g., when compared in volume against that of the discordant disease). Further correlative studies may elucidate the biology underlying discordant PET findings—whether grade migration, development of poorly differentiated neuroendocrine carcinoma, or hypoxia may be contributory to these appearances.

From a technical viewpoint, dual PET analysis presents some difficulties. Typically, the 2 scans are completed on separate days, with the intervening (and unstandardized) period introducing an opportunity for tumor growth or shrinkage and anatomic shift. This may complicate accurate colocalization and limit high-quality reporting. Recent advances such as novel radiotracers and whole-body PET may allow for reduced imaging times and reduced injected radiotracer activity and may facilitate same-day scanning. Additionally, the application of artificial intelligence and machine-learning methods to dual PET data may decrease some of these technical difficulties in the future.

Validation of the current findings in a larger prospective study would further strengthen the quality of evidence surrounding discordant lesions. Such a large-scale study will require significant international cooperation, including formal imaging harmonization. However, given the lack of existing methods to identify patients with discordant disease before dual PET imaging, this will likely involve many patients scanned de novo, with its attendant feasibility and cost implications. Although larger-scale retrospective collaborations would be a desirable way to make progress in an uncommon disease, the challenges associated with this approach include the heterogeneity of practice patterns across different jurisdictions, the differential cost of PET (particularly dual PET), and the privacy issues related to data sharing. Finally, an in-depth quantitative analysis of imaging characteristics, perhaps assisted by machine learning, may yield insights into potential biomarkers. Particularly, further defining the cohort of patients with concordant [^68^Ga]Ga-DOTATATE and [^18^F]FDG avidity (without any discordant disease) would provide a holistic examination of dual PET expression and further the goal of using dual PET as a virtual biopsy to predict tumor biology and disease course. The advent of multiple potential biomarkers for PET-aided prognostication in NENs (together with the need to integrate both [^68^Ga]Ga-DOTATATE PET and [^18^F]FDG PET metrics) also raises the question of potential statistical collinearity, which will have to be carefully considered in future work that compares candidate biomarkers.

## CONCLUSION

We have identified discordant tumor volume as a potential novel imaging biomarker and have shown that higher discordant tumor volume is associated with poorer prognosis in patients with advanced GEPNENs. This prognostication may be used to guide treatment choice. Patients with lower discordant tumor volumes (particularly those with only 1–2 sites of discordant disease) could be considered for ablative approaches for discordant disease sites while simultaneously proceeding with traditional modalities (such as peptide receptor radionuclide therapy) for the bulk of their [^68^Ga]Ga-DOTATATE–avid tumor burden. Despite the challenges associated with a prospective study to validate this biomarker, we hope that the coming postpandemic years will usher in a new era of international collaboration to yield further progress in this challenging disease and ultimately to optimize the outcomes of patients affected by NENs.

## DISCLOSURE

David Chan was funded by the National Health and Medical Research Council of Australia (GNT1175788) and also reports honoraria from Ipsen, Novartis, and Camurus and research support from EMD Serono, outside the submitted work. Gopinath Gnanasegaran reports personal fees from Advanced Accelerator Applications, a Novartis company, and received honoraria for lectures outside the submitted work. Nick Pavlakis reports personal fees from AstraZeneca, Bayer, Boehringer Ingelheim, Merck Serono, Ipsen, Amgen, Bayer Healthcare Pharmaceuticals, Bristol-Myers Squibb, Eli Lilly, Pfizer, Novartis, Roche Pharma AG, Merck, Baxalta, and Specialised Therapeutics, outside the submitted work. Martyn Caplin reports personal fees from Ipsen, Novartis, AAA, Lexicon, and Pfizer and grants from AAA and Ipsen, outside the submitted work. Christos Toumpanakis reports personal fees from Ipsen, Novartis, and AAA and education grants from Ipsen, Novartis, and AAA, outside the submitted work. No other potential conflict of interest relevant to this article was reported.

## Notes

KEY POINTS **QUESTION:** What is the prognostic impact of discordant tumor volume in patients with advanced GEPNENs imaged with dual [^68^Ga]Ga-DOTATATE and [^18^F]FDG PET? **PERTINENT FINDINGS:** This retrospective multicenter study of 44 patients with discordant ([^18^F]FDG-avid/non–[^68^Ga]Ga-DOTATATE–avid) disease showed that those with low discordant tumor volume had statistically better overall survival than those with high discordant tumor volume on univariate analysis. **IMPLICATIONS FOR PATIENT CARE:** Discordant tumor volume may be a prognostic biomarker in patients with advanced GEPNENs.
